# Hand-rolled cigarette smoking patterns compared with factory-made cigarette smoking in New Zealand men

**DOI:** 10.1186/1471-2458-9-194

**Published:** 2009-06-18

**Authors:** Murray Laugesen, Michael Epton, Chris MA Frampton, Marewa Glover, Rod A Lea

**Affiliations:** 1 Health New Zealand Ltd, Christchurch 8082, New Zealand; 2 Canterbury Respiratory Research Group; Department of Medicine, University of Otago, Christchurch, New Zealand; 3 Auckland Tobacco Control Research Centre, School of Population Health, University of Auckland, Auckland, New Zealand; 4 Environmental Science and Research, Kenepuru Drive, Porirua, Wellington region, New Zealand

## Abstract

**Background:**

Roll-your-own (RYO) cigarettes have increased in popularity, yet their comparative potential toxicity is uncertain. This study compares smoking of RYO and factory-made (FM) cigarettes on smoking pattern and immediate potential toxicity.

**Methods:**

At a research clinic, 26 RYO and 22 FM volunteer male cigarette smokers, (addicted and overnight-tobacco-abstinent) each smoked 4 filter cigarettes, one half-hourly over 2 hours, either RYO or FM according to usual habit, using the CReSSMicro flowmeter. First cigarette smoked was their own brand. Subsequent cigarettes, all Holiday regular brand, were RYOs (0.5 g tobacco with filter), or FM with filter. Cravings on 100 mm visual analogue scale, and exhaled carbon monoxide (CO) were measured before and after each cigarette smoked.

**Results:**

Smokers reported similar daily cigarette consumption (RYO 19.0, FM 17.4, p = 0.45), and similar time after waking to first cigarette. (RYO 6.1 minutes, FM 8.6 minutes, p = 0.113). First cigarette's RYO tobacco (0.45 g) weighed less than for FM (0.7 g, p < 0.001); less tobacco was burnt (0.36 g, FM 0.55 g, p < 0.001) but smoking patterns were no different. RYO smokers smoked subsequent cigarettes more intensively; inhaled 28% more smoke per cigarette (RYO 952 mL, FM 743 mL, p = 0.025); took 25% more puffs (RYO 16.9, FM 13.6, p = 0.035); puffed longer (RYO 28 seconds, FM 22 seconds, p = 0.012), taking similar puffs (RYO 57 mL, FM 59 mL). Over four cigarettes, RYOs boosted alveolar CO (RYO 13.8 ppm, FM 13.8 ppm), and reduced cravings (RYO 53%, FM 52%) no differently from FM cigarettes.

**Conclusion:**

In these smokers, RYO smoking was associated with increased smoke exposure per cigarette, and similar CO breath levels, and even with filters is apparently no less and possibly more dangerous than FM smoking. Specific package warnings should warn of RYO smoking's true risk. RYOs are currently taxed much less than FM cigarettes in most countries; similar harm merits similar excise per cigarette.

## Background

Hand-rolled (RYO) cigarettes account for a significant proportion of cigarette smokers in industrialised nations. As the International Tobacco Control survey has shown, of smokers in the United Kingdom, Australia, Canada and USA in 2002, any use of RYOs was reported by 28, 24, 17 and 7%, exclusive use by 17, 9, 7 and 1%, and mixed use by 12, 15, 10 and 6% of smokers respectively [1]. Even more markedly, among New Zealand smokers in that same year, any, exclusive or mixed use of RYO cigarettes accounted for 66%, 37% and 19% respectively, and accounted for 31% of all tobacco used [2,3].

RYO smokers were twice as likely to believe RYO cigarettes posed less risk compared with factory-made (FM) cigarettes [1]. Some smokers obviously enjoy the ritual of rolling a cigarette. Others aware they use less tobacco in smoking RYOs, may believe that RYO cigarettes are somehow safer. Others assume (mistakenly [3]) that RYO cigarettes contain less additives. Yet studies of smoking machine emissions show much higher tar levels in RYO smoke [4-6]. Furthermore, the Norwegian Cancer Registry found that RYO smoking incurs a lung cancer rate almost twice as high as in FM smokers, among 26,000 men and women followed for 28 years [7]. Cigarette emission studies, however, do not allow for the different way in which RYO cigarettes must be smoked. RYO cigarettes are more difficult to keep alight, containing 20% moisture, by comparison with FM cigarettes which contain 13.5% moisture [3]. RYO cigarette papers contain no citrate accelerant and self-extinguish if not puffed [8], and are often re-lit. For economic reasons also, RYO smokers roll a thin cigarette and then smoke it to extract maximum satisfaction.

In order to compare toxicity of RYO and FM cigarettes it is important to allow for product and cost differences influencing human puffing patterns. This study was designed to explore smoking topography (puffing patterns), and immediate toxicity, of RYO cigarettes and FM cigarettes in New Zealand men.

## Methods

The study was undertaken in smoking men living in the city of Christchurch, in Canterbury, New Zealand. Christchurch has a population of 348,435, of a total population of 374,715 in the Canterbury district health board (DHB) area [9]. In the DHB's area, 20.2% of men were regular cigarette smokers, comprising 36,522 in total [9]. 6% of men in Canterbury identify as Maori, compared with 11.3% nationwide. Nationwide, 20.3% of European men and 38.5% of Maori men smoked [10]. Nationwide, RYO was the preferred cigarette type of 54% of European male and 61% of Maori male smokers, and of 49% of male and of 47% of female smokers[11].

Male volunteer smokers aged 18 and over were recruited by newspaper advertisements (15 attending out of 68 callers), from a general practice smoking register (3 attended out of 44 smokers sent a letter by their general practitioner); and a further 30 attended after phoning 821 recent callers to a national toll-free quitting helpline who had given prior consent to be contacted. Many on the helpline list had already recently quit smoking, making them ineligible. In total 933 subjects, comprising 2.6% of regular male smokers in Christchurch were contacted. Eligible smokers had to currently smoke at least five cigarettes a day, smoke their first cigarette of the day within half an hour of waking, and be willing to attend the clinic for the first two hours of the working day on a weekday. Smokers with recent or unstable cardiovascular or respiratory disease were excluded, along with any who had recently used non-cigarette nicotine or tobacco, or illegal drugs. Nine were booked to attend but missed their clinic appointment. In total 48 smokers were accepted into the study and allocated into one of two groups, according to whether their predominant cigarettes smoked were RYO or FM.

Smokers attended the research clinic early in the morning, before the first cigarette of the day. Morning abstinence was confirmed from exhaled CO by MicroCO meter (Cardinal Health, Chatham, Kent UK). A breath CO > 15 ppm necessitated rescheduling of the appointment. A questionnaire assessed smoking patterns, using the Heavy Smoking Index for nicotine addiction [12]. Assessment of socio-economic deprivation was made using the NZ Deprivation Index, a composite decile scale based on Census derived data related to the subject's address (1 indicates least deprivation, 10 the most deprivation) [13].

FM smokers smoked their own brand for the first cigarette of the day, and subsequently smoked three further cigarettes provided by the investigators (Holiday FM special filter regular cigarettes – 84 mm in length, 8 mm diameter, including a cellulose acetate filter 20 mm in length).

RYO smokers smoked RYO cigarette tobacco of their usual brand and amount for the first cigarette, with filter, and subsequently rolled and smoked Holiday regular cigarette tobacco for three further cigarettes with filter. In rolling these subsequent RYO cigarettes, each volunteer used 0.5 g of tobacco supplied and weighed by the investigators, using Rizla paper (70 mm length, 30 mm wide up to the adhesive edge) with a Boomerang regular cellulose acetate filter (8 mm diameter, 15 mm in length).

A total of four cigarettes were smoked by each volunteer, half-hourly over two hours. Each unburnt cigarette (RYO or FM) was weighed by electronic balance, accurate to 1 mg (Acculab analytic balance, ALC 150.3, Acculab Asia-Pacific. Kowloon, Hong Kong). The burnt butt was weighed after ash removal. Net tobacco weight was calculated before and after smoking by deduction of the weight of filters and paper.

The smoking pattern of each cigarette smoked was measured by the CReSSMicro (Pocket) cigarette holder-flowmeter (Borgwaldt KC GmbH, Hamburg, Germany) which generates data on the date and time of cigarette insertion and removal, puff volume, puff duration, inter-puff interval (IPI), puff count, and peak inspiratory flow rate during a puff [14]. Before and after each cigarette, the smoker assessed his own cravings in response to the question "Right now, how much do you want a cigarette?" The smoker marked a 100 mm Visual Analogue Scale (VAS), with "Not at all" rated 0 at one end of the line, and "Extremely" rated 100 at the other end. The VAS was previously validated against arterial serum nicotine [15]. Exhaled CO was measured in expired breath immediately before, and five minutes after finishing each cigarette [16]. Outcome measures studied between groups included total puff volume per cigarette, number of puffs per cigarette, and volume smoked per puff. For each group and between groups, the first cigarette was compared to the subsequent three cigarettes. Immediate toxicity was inferred from CO boost per cigarette.

The study was approved by the Upper South A Regional Ethics committee of the Ministry of Health, and by the University of Otago. All volunteers provided written informed consent prior to commencement, and were paid in vouchers on completion.

### Statistics

The demographic and smoking habits of the two study groups were compared statistically using Chi-squared tests for categorical measures and independent t-tests for continuous measures. Smoking exposure measures and CO boosts with and without adjustment for weight of tobacco smoked were compared between the RYO and FM cigarette smokers using independent t-tests. A two-tailed p-value < 0.05 was taken to indicate statistical significance. There were no existing data on CO or smoke inhalation levels upon which to base a power calculation, but a minimum of 20 participants was sought for each of the smoker groups.

## Results

### The study population

48 men took part in the study (26 RYO cigarette smokers, 22 FM cigarette smokers). The demographics of the study population are shown in Table 1. There was no significant difference between the two groups, except that RYO smokers were significantly younger than FM smokers (37 years versus 47 years, p = 0.026). Seven out of the 48 identified themselves as Maori, the rest were of European origin. The subjects' mean Heavy Smoking Index (HSI) was 3.67: an HSI of 4 indicates high nicotine dependence, equivalent to a Fagerstrom Score of 6 [12]. RYO and FM smokers reported similar daily cigarette consumption (RYO 19.0, FM 17.4, p = 0.45), and similar time after waking to first cigarette. (RYO 6.1, FM 8.6 minutes, p = 0.113).

**Table 1:** The study population

**(Males-only study)**	**Smokers of Roll-your-own (RYO) cigarettes n = 26**	**Smokers of Factory-made (FM) cigarettes n= 22**	**All** **n = 48**	**p values for RYO vs FM**
Average age, years (sd)	39 (13)	47 (13)	43 (13)	0.026

Maori, n	5	2	7	
				0.43#
European, n	21	20	41	

Height cms (sd)	175.2 (9.3)	177.5 (7.9)	176.3 (8.7)	0.372

Weight kg (sd)	85.5 (28.2)	93.6 (28.7)	89.24 (28.5)	0.333

NZ Deprivation index of census unit area* average decile (sd)	5.6 (2.7)	4.7 (3.4)	5.2 (3.0)	0.312

Time to first cigarette of the day, minutes (sd)	6.06 (5.71)	8.64 (5.27)	7.24 (5.61)	0.113

Cigarettes per day (sd)	19.0 (6.8) RYO and 0.6 (1.18) FM	17.4 (7.5) FM and 0.1 (0.4) RYO	18.63 (7.00)	0.454

Heavy smoking index (sd)	3.77 (1.27)	3.55 (0.91)	3.67 (1.12)	0.495

Cravings before 1^st ^cigarette of the day. VAS score (sd) (where 100 = extreme craving)	62.9 (24.2)	57.8 (23.9)	60.6 (23.9)	0.475

*Higher score indicates higher deprivation, n = 20 for FM group.

p values are based on Chi-squared tests for categorical measures and independent t-tests for continuous measures.

# No significant difference in RYO to FM proportions in either Maori or European.

### Smoking of the first cigarette

RYO first cigarettes used less RYO tobacco (455 mg), significantly less than first FM cigarettes (714 mg, p < 0.001); and 35% less RYO tobacco was burnt (RYO 361 mg, FM 552 mg, p < 0.001) yet smoking patterns were no different statistically between RYO and FM smokers.

Cravings decreased significantly in both groups after the first cigarette; and reduced to a similar degree between RYO smokers and FM smokers. There was no difference in CO boost between groups after the first cigarette, even when adjusted for weight of tobacco burnt (data not shown).

### Smoking of subsequent cigarettes

For subsequent cigarettes (Table 2), RYO cigarettes, though containing less tobacco, were smoked more intensively. RYO smokers spent more time puffing (RYO 28 seconds per cigarette, FM 22 seconds, p = 0.012); they inhaled smoke 25% more often (RYO 17 puffs, FM 14 puffs, p = 0.035). There was no difference in depth of puff between groups. Smoke inhalation per cigarette was 28% higher in the RYO group (RYO 952 mL, FM 743 mL, p = 0.025). The reduction in craving after these subsequent cigarettes was less than after the first cigarette of the day; there was however no difference in craving reduction between the two groups. There was no difference in CO boost between groups after any of the subsequent cigarettes, but CO boost when adjusted for tobacco smoked, was significantly greater for the RYO group (RYO 10.8 ppm/g, FM 7.6 ppm/g, p = 0.02).

**Table 2:** Smoke exposure measures and CO boost, by type of cigarette smoked; 2^nd^, 3^rd^, 4^th ^cigarettes averaged

Averages, per cigarette (standard deviations)	**Smokers of Roll-your-own (RYO) cigarettes** **n = 26**	**Smokers of Factory-made (FM) cigarettes** **n = 22**	**p value for the RYO – FM difference of means**
Weight of tobacco mg per cigarette	496.5 (7.47)	695.3 (15.0)	<0.001*

Net weight of tobacco burnt, own cigarette brand, mg	400.3 (48.4)	552.4 (87.8)	<0.001*

CO increase per cigarette, ppm	4.37 (2.06)	4.24 (2.52)	0.846

CO ppm increase per g of tobacco burnt;	10.82 (4.98)	7.60 (4.26)	0.021*

Total smoke inhaled per cigarette (total puff volume) mL	952.1 (387.7)	742.6 (188.8)	0.025*

Smoke inhaled in mL, per g of tobacco burnt	2392.6 (1013.0)	1356.0 (321.4)	<0.001*

Smoke inhaled per day mL #	18803 (13030)	13242 (6738)	0.077

Puffs per cigarette, count	16.9 (5.2)	13.6 (5.4)	0.035*

Smoke volume per puff, mL	57.3 (17.1)	58.9 (16.8)	0.744

Smoke flow mL/second	33.9 (10.4)	35.2 (9.3)	0.663

Smoke peak flow ml/second	51.0 (18.2)	51.4 (14.0)	0.928

Duration per puff seconds	1.72 (0.40)	1.77 (0.45)	0.664

Average inter-puff interval, seconds	16.67 (9.44)	18.67 (7.11)	0.419

Time puffing per cigarette, seconds	28.4 (9.1)	22.4 (5.9)	0.012*

Puffing as % of time smoking	11.67 (4.51)	10.24 (3.58)	0.235

Minutes smoking, per cigarette	4.29 (0.99)	3.86 (0.96)	0.139

Cravings from after 1^st ^to after 4^th ^cigarette	24.2 (28.2) to 10.1 (21.6)	19.8 (25.2) to 6.1 (9.1)	0.332

*p values (statistically significant at p =< 0.05) are based on Chi-squared tests for categorical measures and independent t-tests for continuous measures.

# Product of smoke volume per cigarette and number of cigarettes per day (from Table 1).

## Discussion

This study demonstrates that smoking patterns of RYO smokers in New Zealand men are different from smokers of FM cigarettes. Whilst the amount of tobacco incorporated into a RYO cigarette is less, the pattern of smoking, particularly for later cigarettes in the day, increases inhalation of tobacco smoke. The study was not able to demonstrate differences in immediate toxicity per cigarette, as measured by CO boost, but CO boost was higher per gram of RYO tobacco smoked or burnt than per gram of FM tobacco smoked or burnt. The CO data taken together with the smoke inhalation data, suggest that RYO smoking even with filters, is at least as harmful as FM smoking, and was more harmful per gram of tobacco smoked.

Recruitment was slow and only 5% of smokers contacted attended the clinic, but the recruitment target of at least 20 per smoker group was achieved. Although the total sample size is not large the study was sufficiently powered to detect important differences between the groups as evidenced by the statistically significant results for the smoke exposure and CO boost per gram measures. More subtle differences between the groups, for example time smoking each type of cigarette, will require larger studies to provide conclusive data.

Although RYO smokers tended to be younger, they did not differ significantly from FM smokers as a group on the important variables of time to first cigarette and cigarettes smoked per day, or the deprivation index of their locality. There was no inter-group difference in the smoking pattern of the first cigarette of the day between RYO smokers and FM smokers. One explanation could be that the significant level of craving in both groups following overnight abstinence led to similarly intensive smoking. Also, the first cigarette of the study day was the first exposure of the smokers to the CReSSMicro device, which might have altered initial smoking patterns. Subsequent smoking topography might be less affected by the device, with the smokers becoming more accustomed to the device and the environment. However, we confirm Shahab's findings from the UK that RYO smokers' puffing behaviour can be measured conveniently with the CReSSMicro [17].

This study cannot discriminate whether individual smokers would smoke RYO cigarettes differently from FM cigarettes, that is, whether topography and toxicity differences relate to the smoker or the cigarette type. A within-smoker study switching between RYO and FM cigarettes would better elucidate smoking differences due to type of cigarette.

This study was restricted to men. For women, a larger study would be needed, to allow for the variably increased nicotine metabolism in women, due to the effects of oestrogen [18].

A recent study of smoke machine emissions compared Holiday RYO versus Holiday FM cigarettes, and found higher tar and nicotine yields from RYO cigarettes. Correcting for nicotine, emissions from RYO cigarettes were higher for tar but lower for CO, acrolein and some carcinogenic gases [6]. In this study we preferred to measure the per cigarette increase in alveolar CO, (the CO boost) a more direct measure of harm to the smoker; we found that the CO boost was similar for RYO and FM smoking.

## Conclusion

In New Zealand, comparatively high excise rates per gram of tobacco over many years have encouraged smokers to hand roll cigarettes thin and pay less tax. Thus excise increases have perversely encouraged cheaper smoking rather than quitting. RYO smokers today not only use less tobacco (0.5 g or less) they also waste less smoke as sidestream smoke, inhaling more for themselves, and so not surprisingly, clinical measurement suggests that RYO smoking is at least as harmful as FM smoking.

RYO smokers use less tobacco but smoke more intensively, take more puffs, inhale more smoke per cigarette and for longer, and absorb equivalent concentrations of CO.

Placement of specific warnings on RYO packaging and an increase in the excise rate for loose cigarette tobacco so that the excise per RYO cigarette equals the excise per FM cigarette, would alert smokers to the fact that RYO and FM smoking are similarly hazardous.

## Competing interests

The authors declare that they have no competing interests.

## Authors' contributions

ML conceptualised the study and arranged funding; ML, ME, MG and RL developed and managed the study, ML, ME and CF analysed the results and wrote the paper. All authors read and approved the final manuscript.

## Pre-publication history

The pre-publication history for this paper can be accessed here:


